# Two distinct *Dehalobacter* metagenome-assembled genomes from anaerobic chloroform and dichloromethane degrading consortia

**DOI:** 10.1128/mra.00803-24

**Published:** 2024-09-24

**Authors:** Olivia Bulka, Elizabeth A. Edwards

**Affiliations:** 1 Department of Chemical Engineering and Applied Chemistry, University of Toronto, Toronto, Ontario, Canada; Montana State University, Bozeman, Montana, USA

**Keywords:** *Dehalobacter*, genome, metagenome-assembled genome, metagenome, bioremediation, chloroform, dichloromethane

## Abstract

Here we present two metagenomes and two *Dehalobacter* metagenome-assembled genomes from subcultures of an anaerobic chloroform and dichloromethane degrading microbial community used for bioremediation. Our objective was to assemble and curate the genome(s) of *Dehalobacter*, key biodegraders in the culture, through repeated sequencing and joint assembly with previous datasets.

## ANNOUNCEMENT

**Fig 1:**
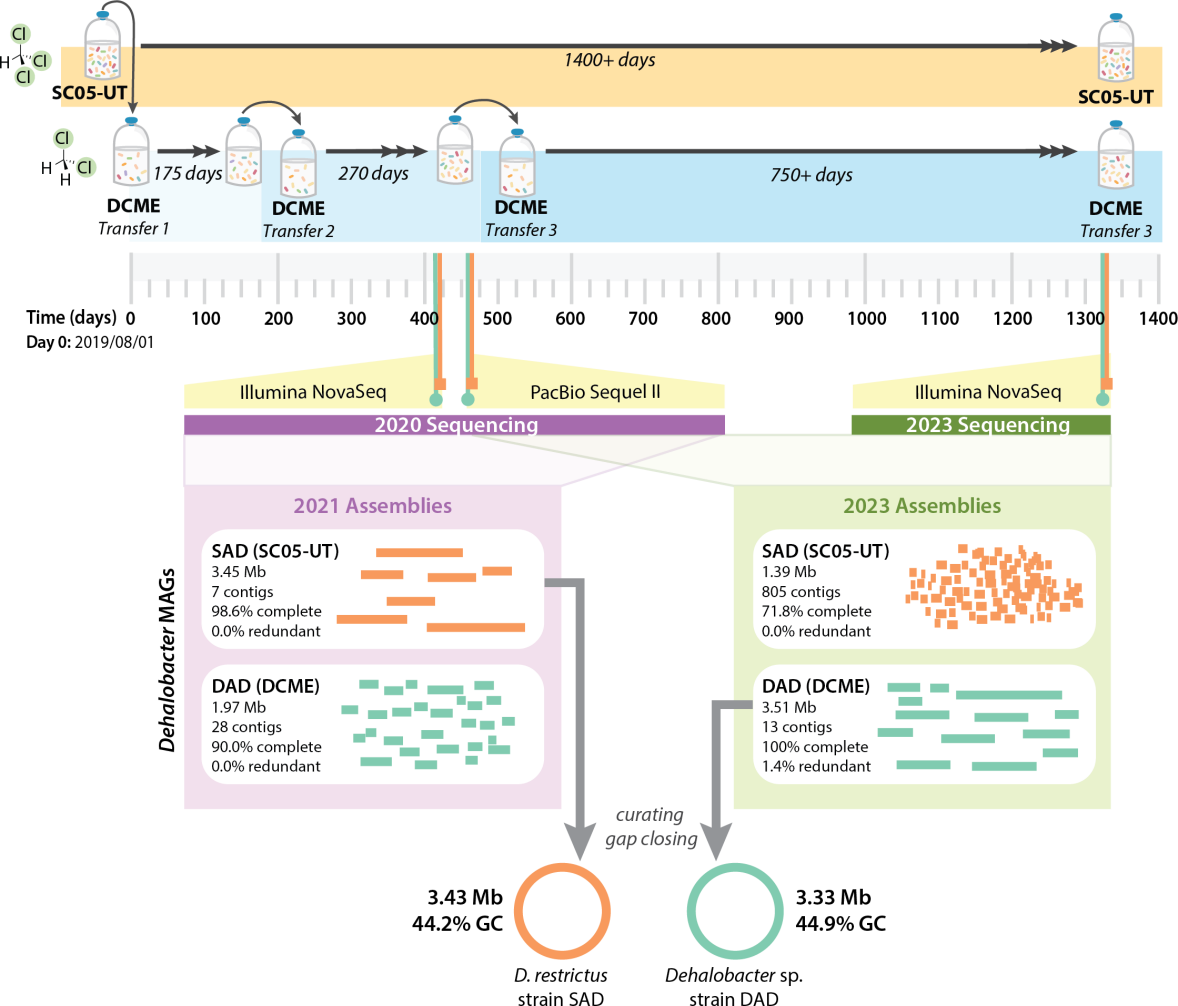
Summary of experimental setup, sequencing, and assembly of *Dehalobacter* MAGs. DNA samples are noted on the timeline (SC05-UT: orange squares, DCME: green circles) and labeled with the type of sequencing. Sequencing/assembly performed in 2021 are marked with purple [further described in reference (4)], and sequencing/assembly described in this study are highlighted in green. Stats from each *Dehalobacter* MAG in each metagenome assembly are compared in the purple (2021) and green (2023) panels, with gray arrows denoting which was selected for further curation and gap closing. *Dehalobacter restrictus* strain SAD (from SC05-UT): orange genome, *Dehalobacter* sp. strain DAD (from DCME): green genome.

SC05 is a bioremediation culture originally sampled from contaminated groundwater in California (also known as KB-1 Plus CF; SiREM, Guelph, ON). SC05 dechlorinates chloroform (CF) completely to carbon dioxide and hydrogen via dichloromethane (DCM) (1). The SC05-UT subculture has been fed CF without an exogenous electron donor since 2018; it dechlorinates CF using electrons produced from DCM mineralization. The DCME subculture has been fed DCM alone since 2019. Both subcultures were maintained continuously (~1 mM CF or DCM [aqueous] biweekly-monthly) to enrich microbes performing key biotransformation steps in the culture: CF dechlorination and DCM mineralization (Fig. 1) (2). Both steps were linked to *Dehalobacter* via qPCR (2) and proteogenomics (3); *Dehalobacter* expressed the necessary proteins for both biotransformations (3). The original assemblies [described in our related work (4)] were unable to resolve complete genomes due to uneven coverage from PCR amplification before sequencing. Here, we describe additional sequencing to determine variation in the *Dehalobacter* populations of each SC05 subculture.

SC05-UT and DCME were sampled in March 2023 (30 mL), and DNA was extracted using the DNeasy PowerSoil kit (Qiagen, Hilden, Germany) prior to sequencing by the Genome Quebec Innovation Centre (Montréal, QC, Canada). Libraries were prepared using the NxSeq AmpFREE Low DNA Library Kit (Lucigen, Middleton, WI) with an average insert size of 250 nt. Illumina NovaSeq 6000 sequencing generated 150 nt paired-end reads (Table 1), which were quality controlled using FastQC v0.11.9 (5). The first three bases were trimmed using Trimmomatic v0.39 (6). Each set of Illumina reads was pooled with previously sequenced PacBio reads from the same subculture [SC05-UT: SRR25941764, DCME: SRR25941763 (4)] for co-assembly using hybridSPAdes (SPAdes v3.15.0, python v3.6.12) (7). All software used default parameters. Additional quality control, mapping, and binning of contigs >1 kb were performed within Anvi’o v7 (8–10), using MetaBAT2 v2.15 (11), MaxBin v2.2.7 (12), and CONCOCT v1.1.0 (13). DAS Tool v1.1.2 (14) was used to dereplicate and select MAGs >50% complete and <30% redundant. Taxonomy of the 106 resulting MAGs (mean completion: 87.5%, mean redundancy: 5.0%) was determined in Anvi’o using DIAMOND v0.9.14 (15) and the GTDB v95.0 (16). Assembly statistics were generated with anvi-summarize in Anvi’o (8–10) (Table 1).

**TABLE 1:** Summary of metagenomes and reads reported in this study

Assembly	Number of reads (×2)	Assembly size (Mb)	Number of contigs	*N*_50_ (bp)	Accession (Paired-end reads: SRA, Assemblies: Genbank)
SC05-UT metagenome	89,451,995	241.2	29,796	28,488	SRR27458442, JBAWSR000000000
DCME metagenome	84,638,297	240.2	28,400	43,688	SRR27458441, JBAWSS000000000

One *Dehalobacter* MAG was produced from each assembly, which were compared to those from prior sequencing for dereplication (4) (Fig. 1). The best MAG from each subculture was manually curated in Geneious v8.1.8 (17). Gapfilling was performed using PacBio HiFi reads from each culture. Adjoining contigs were connected using the Geneious *de novo* assembly tool, and ambiguities were resolved using the Geneious read mapper (17). Each MAG was oriented at the *dnaA* gene, using iRep to calculate GC skew (18). The closed MAGs were named *Dehalobacter restrictus* strain SAD (SC05-UT–assembled *Dehalobacter*; 3.43 Mb, 44.2% GC, mean coverage: 3519.8) and *Dehalobacter* sp. strain DAD (DCME–assembled *Dehalobacter*; 3.33 Mb, 44.9% GC, mean coverage: 943.4). Both were annotated using PGAP v6.7 (19), predicting 3,394 and 3,311 genes in strains SAD and DAD, respectively. A detailed comparison of these *Dehalobacter* strains and their roles in SC05 is forthcoming.

## Data Availability

All reads and assemblies are available on NCBI under BioProject PRJNA1013980. These *Dehalobacter* metagenome-assembled genomes are deposited under accession numbers CP148031 (DAD) and CP148032 (SAD). The Whole Genome Shotgun projects have also been deposited in GenBank under the accession no. JBAWSR000000000 (SC05-UT) and JBAWSS000000000 (DCME). The version described in this paper is the first version, JBAWSR010000000 and JBAWSS010000000. Illumina paired reads have been deposited in the Sequence Read Archive under accession numbers SRR27458442 (SC05-UT) and SRR27458441 (DCME). A summary of the quality statistics and taxonomy of all draft MAGs is available on FigShare at 10.6084 /m9.figshare.25843750. Fasta files of all draft MAGs in the DCME and SC05-UT metagenomes are available at 10.6084 /m9.figshare.25843399 and 10.6084 /m9.figshare.25843396, respectively.

## References

[1] Wang H, Yu R, Webb J, Dollar P, Freedman DL. 2022. Anaerobic biodegradation of chloroform and dichloromethane with a Dehalobacter enrichment culture. Appl Environ Microbiol 88:e0197021. doi:10.1128/AEM.01970-21 34936839 PMC8863061

[2] Bulka O, Webb J, Dworatzek S, Mahadevan R, Edwards EA. 2023. A multifunctional Dehalobacter? Tandem chloroform and dichloromethane degradation in a mixed microbial culture. Environ Sci Technol 57:19912–19920. doi:10.1021/acs.est.3c06686 37962431

[3] Bulka O, Picott K, Mahadevan R, Edwards EA. 2024. From mec cassette to rdhA: a key dehalobacter genomic neighborhood in a chloroform and dichloromethane–transforming microbial consortium. Appl Environ Microbiol 90:1–24. doi:10.1128/aem.00732-24 PMC11218628 38819127

[4] Bulka O, Edwards EA. 2024. Metagenomic sequences from anaerobic chloroform and dichloromethane degrading microbial communities. Microbiol Resour Announc 13:e0039124. doi:10.1128/mra.00391-24 38949307 PMC11324032

[5] Andrews S, Krueger F, Seconds-Pichon A, Biggins F, Wingett S. 2010. FastQC. A quality control tool for high throughput sequence data. Available online at: http://www.bioinformatics.babraham.ac.uk/projects/fastqc.

[6] Bolger AM, Lohse M, Usadel B. 2014. Trimmomatic: a flexible trimmer for Illumina sequence data. Bioinformatics 30:2114–2120. doi:10.1093/bioinformatics/btu170 24695404 PMC4103590

[7] Antipov D, Korobeynikov A, McLean JS, Pevzner PA. 2016. hybridSPAdes: an algorithm for hybrid assembly of short and long reads. Bioinformatics 32:1009–1015. doi:10.1093/bioinformatics/btv688 26589280 PMC4907386

[8] Eren A Murat, Esen ÖC, Quince C, Vineis JH, Morrison HG, Sogin ML, Delmont TO. 2015. Anvi’o: an advanced analysis and visualization platform for ’omics data. PeerJ 3:e1319. doi:10.7717/peerj.1319 26500826 PMC4614810

[9] Eren A.M, Kiefl E, Shaiber A, Veseli I, Miller SE, Schechter MS, Fink I, Pan JN, Yousef M, Fogarty EC, et al.. 2021. Community-led, integrated, reproducible multi-omics with anvi’o. Nat Microbiol 6:3–6. doi:10.1038/s41564-020-00834-3 33349678 PMC8116326

[10] Shaiber A, Willis AD, Delmont TO, Roux S, Chen LX, Schmid AC, Yousef M, Watson AR, Lolans K, Esen ÖC, Lee STM, Downey N, Morrison HG, Dewhirst FE, Mark Welch JL, Eren AM. 2020. Functional and genetic markers of niche partitioning among enigmatic members of the human oral microbiome. Genome Biol 21:292. doi:10.1186/s13059-020-02195-w 33323122 PMC7739484

[11] Kang DD, Li F, Kirton E, Thomas A, Egan R, An H, Wang Z. 2019. MetaBAT 2: an adaptive binning algorithm for robust and efficient genome reconstruction from metagenome assemblies. PeerJ 7:e7359. doi:10.7717/peerj.7359 31388474 PMC6662567

[12] Wu YW, Tang YH, Tringe SG, Simmons BA, Singer SW. 2014. MaxBin: an automated binning method to recover individual genomes from metagenomes using an expectation-maximization algorithm. Microbiome 2:26. doi:10.1186/2049-2618-2-26 25136443 PMC4129434

[13] Alneberg J, Bjarnason BS, de Bruijn I, Schirmer M, Quick J, Ijaz UZ, Lahti L, Loman NJ, Andersson AF, Quince C. 2014. Binning metagenomic contigs by coverage and composition. Nat Methods 11:1144–1146. doi:10.1038/nmeth.3103 25218180

[14] Sieber CMK, Probst AJ, Sharrar A, Thomas BC, Hess M, Tringe SG, Banfield JF. 2018. Recovery of genomes from metagenomes via a dereplication, aggregation and scoring strategy. Nat Microbiol 3:836–843. doi:10.1038/s41564-018-0171-1 29807988 PMC6786971

[15] Buchfink B, Xie C, Huson DH. 2015. Fast and sensitive protein alignment using DIAMOND. Nat Methods 12:59–60. doi:10.1038/nmeth.3176 25402007

[16] Parks DH, Chuvochina M, Waite DW, Rinke C, Skarshewski A, Chaumeil PA, Hugenholtz P. 2018. A standardized bacterial taxonomy based on genome phylogeny substantially revises the tree of life. Nat Biotechnol 36:996–1004. doi:10.1038/nbt.4229 30148503

[17] Kearse M, Moir R, Wilson A, Stones-Havas S, Cheung M, Sturrock S, Buxton S, Cooper A, Markowitz S, Duran C, Thierer T, Ashton B, Meintjes P, Drummond A, Valencia A. 2012. Geneious basic: an integrated and extendable desktop software platform for the organization and analysis of sequence data. Bioinformatics 28:1647–1649. doi:10.1093/bioinformatics/bts199 22543367 PMC3371832

[18] Brown CT, Olm MR, Thomas BC, Banfield JF. 2016. Measurement of bacterial replication rates in microbial communities. Nat Biotechnol 34:1256–1263. doi:10.1038/nbt.3704 27819664 PMC5538567

[19] Tatusova T, DiCuccio M, Badretdin A, Chetvernin V, Nawrocki EP, Zaslavsky L, Lomsadze A, Pruitt KD, Borodovsky M, Ostell J. 2016. NCBI prokaryotic genome annotation pipeline. Nucleic Acids Res 44:6614–6624. doi:10.1093/nar/gkw569 27342282 PMC5001611

